# Retinal tissue hypoperfusion in patients with clinical Alzheimer’s disease

**DOI:** 10.1186/s40662-018-0115-0

**Published:** 2018-08-17

**Authors:** Giovana Rosa Gameiro, Hong Jiang, Yi Liu, Yuqing Deng, Xiaoyan Sun, Bernardo Nascentes, Bernard Baumel, Tatjana Rundek, Jianhua Wang

**Affiliations:** 1 Department of Ophthalmology, Bascom Palmer Eye Institute, University of Miami Miller School of Medicine, 1638 NW 10th Avenue, McKnight Building - Room 202A, Miami, FL 33136 USA; 2 Evelyn F. McKnight Brain Institute, Department of Neurology, University of Miami Miller School of Medicine, Miami, FL USA; 3 Department of Ophthalmology, Third Affiliated Hospital of Nanjing University of Chinese Medicine, Nanjing, China; 4 State Key Laboratory of Ophthalmology, Zhongshan Ophthalmic Center, Sun Yat-sen University, Guangzhou, Guangdong China; 5 School of Nursing and Health Studies, University of Miami, Miami, FL USA

**Keywords:** Clinical Alzheimer’s disease, Retinal tissue perfusion, Blood flow, Retinal tissue volume, Hypoperfusion, Retinal function imager, Optical coherence tomography

## Abstract

**Background:**

It remains unknow whether retinal tissue perfusion occurs in patients with Alzheimer’s disease. The goal was to determine retinal tissue perfusion in patients with clinical Alzheimer’s disease (CAD).

**Methods:**

Twenty-four CAD patients and 19 cognitively normal (CN) age-matched controls were recruited. A retinal function imager (RFI, Optical Imaging Ltd., Rehovot, Israel) was used to measure the retinal blood flow supplying the macular area of a diameter of 2.5 mm centered on the fovea. Blood flow volumes of arterioles (entering the macular region) and venules (exiting the macular region) of the supplied area were calculated. Macular blood flow was calculated as the average of arteriolar and venular flow volumes. Custom ultra-high-resolution optical coherence tomography (UHR–OCT) was used to calculate macular tissue volume. Automated segmentation software (Orion, Voxeleron LLC, Pleasanton, CA) was used to segment six intra-retinal layers in the 2.5 mm (diameter) area centered on the fovea. The inner retina (containing vessel network), including retinal nerve fiber layer (RNFL), ganglion cell-inner plexiform layer (GCIPL), inner nuclear layer (INL) and outer plexiform layer (OPL), was segmented and tissue volume was calculated. Perfusion was calculated as the flow divided by the tissue volume.

**Results:**

The tissue perfusion in CAD patients was 2.58 ± 0.79 nl/s/mm^3^ (mean ± standard deviation) and was significantly lower than in CN subjects (3.62 ± 0.44 nl/s/mm^3^, *P* <  0.01), reflecting a decrease of 29%. The flow volume was 2.82 ± 0.92 nl/s in CAD patients, which was 31% lower than in CN subjects (4.09 ± 0.46 nl/s, P <  0.01). GCIPL tissue volume was 0.47 ± 0.04 mm^3^ in CAD patients and 6% lower than CN subjects (0.50 ± 0.05 mm^3^, *P* < 0.05). No other significant alterations were found in the intra-retinal layers between CAD and CN participants.

**Conclusions:**

This study is the first to show decreased retinal tissue perfusion that may be indicative of diminished tissue metabolic activity in patients with clinical Alzheimer’s disease.

## Background

Dementia is a group of symptoms associated with memory loss mostly in the elderly, impacting not only patients but also their families, caregivers, communities, and the society. There are more than 50 million people suffering from dementia, and nearly 10 million new cases are being diagnosed every year [1]. Clinical Alzheimer’s disease (CAD) is the most common form of dementia. Accumulated evidence from epidemiological, pharmacotherapeutic, and neuroimaging studies suggest that vascular alterations contribute to AD [2]. Furthermore, global and focal cerebral hypoperfusion measured by MRI and transcranial Doppler (TCD) are often present in patients with AD [3–5].

**Fig. 1:**
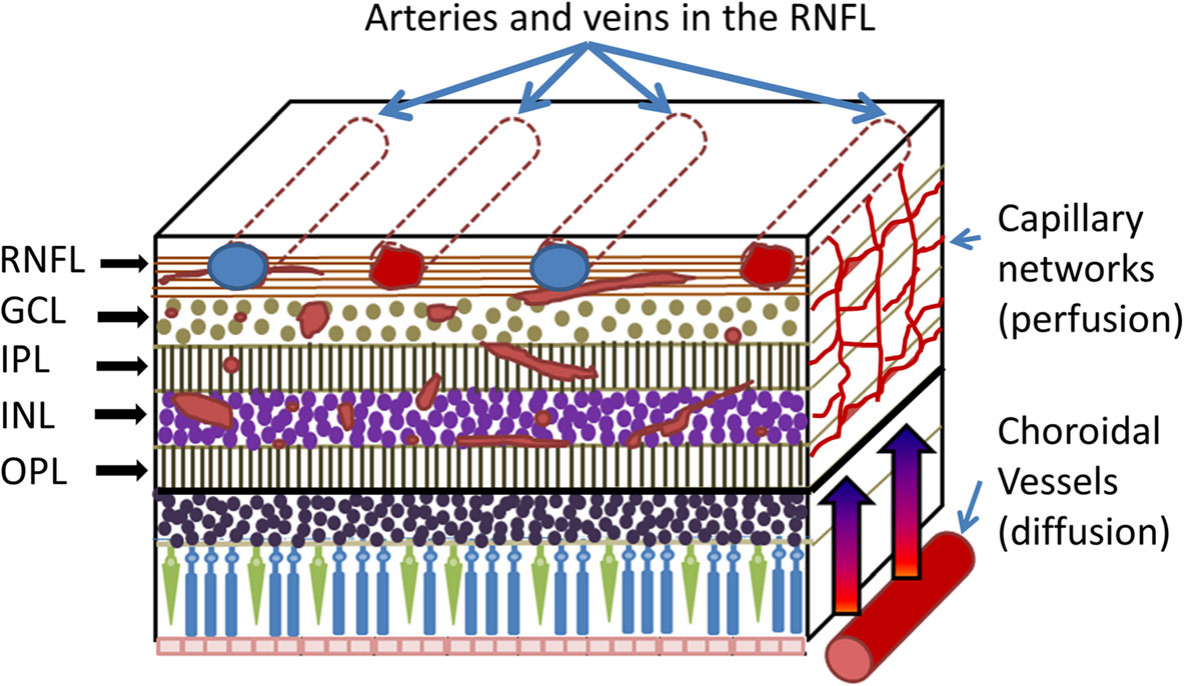
Schematic of retinal blood supply. The capillary networks within the RNFL, GCL, IPL, INL and OPL perfuse the inner retina. However, according to a recent study using optical coherence tomography [8], since there are no vessels in the outer retina, choroidal vessels perfuse this region through diffusion. RNFL: retinal nerve fiber layer; GCL: ganglion cell layer; IPL: inner plexiform layer; INL: inner nuclear layer; OPL: outer plexiform layer

The eye is regarded as a window to the brain, which may facilitate the detection of early AD-related vascular impairment [6, 7]. The choroidal vessels perfuse the outer retina by diffusion because there is no capillary network in the outer retina (Fig. 1) [8], whereas the branches of central retinal arteries and veins lie within the retinal nerve fiber layer, and their capillary networks from the interior limiting membrane to the outer nuclear layer supply the inner retina [8]. This unique vessel distribution and blood supply provide an opportunity for quantitative analysis of tissue perfusion in the retina, thus reflecting cerebral perfusion status.

The Retinal Function Imager (RFI) can distinctly measure blood flow velocity and blood flow rate of arterioles and venules in the retinal nerve fiber layer (RNFL). The vessels supplying the fovea have a unique layout, in which the terminal branches of the superior temporal and inferior temporal arteries point towards the center of the fovea and stagger with the retinal venules. This characteristic vessel distribution makes it possible to quantitatively measure the blood flow supplying the macular area. Our previous studies successfully determined macular flow by measuring the flow in each branch of the vessel crossing a circle centered on the fovea [7, 9]. Decreased macular blood flow volume is reported in patients with AD [7]. Meanwhile, the loss of retinal neural tissue, reported as a thinning of the retinal ganglion cell layer (GCL) and RNFL, measured by optical coherence tomography (OCT), is evident [10–13]. The inner retina is supplied by the central retinal artery [8]. Hence, the loss of RNFL and ganglion cell inner plexiform layer (GCIPL), located in the inner two-thirds of the retina, may relate to the changes in retinal blood supply, and also co-exist as an alteration of tissue perfusion. Tissue perfusion is typically quantified by measuring the volume of blood passing through the vascular network in a given volume of the tissue over a certain amount of time [14]. Although previous studies showed a decline of macular blood flow volume [7] and retinal tissue [10, 11] in patients with AD compared to cognitively normal (CN) controls, it remains unknown whether retinal tissue hypoperfusion indeed occurs in AD patients. The goal of the present study was to determine retinal tissue perfusion in the macula of CAD patients.

## Methods

The study was approved by the institutional review board for human research at the University of Miami, and each subject signed an informed consent form. All patients and control subjects were treated in accordance with the tenets of the Declaration of Helsinki. The patients were recruited at the Division of Cognitive Disorders of the Department of Neurology at the University of Miami, and the diagnoses were made according to the National Institute on Aging-Alzheimer’s Association (NIA-AA) criteria [15]. The AD diagnoses were discussed and confirmed in a group consensus conference including neurologists, psychiatrists, and neuropsychologists. Cognitively normal (CN) controls were recruited from subjects who received annual eye examinations or the family members of the patients at the Bascom Palmer Eye Institute, University of Miami.

A total of 24 AD patients and 19 CN controls with a similar age range were recruited (Table 1). Subjects with other cardiovascular diseases or systemic diseases such as a history of stroke, coagulopathy, and uncontrolled hypertension and diabetes were excluded from the study. As ocular diseases may affect the results, subjects with high refractive errors of more than + 6.0 or − 6.0 diopters, age-related macular degeneration, cystic macular edema, severe cataracts, diabetic retinopathy, glaucoma, and cornea disease were excluded. As the RFI utilizes a fundus camera, severe cataract can affect the image acquisition therefore, patients with severe cataract were excluded. In this study, there were 9 patients with intraocular lenses implanted in the AD group and 0 subject in the control group. In addition, retinal blood flow was visualized on RFI acquired sessions, which ensured that blood flow was measurable.

**Table 1:** Demographics and clinical manifestations of study patients and normal subjects

	AD (*N* = 24)	CN (*N* = 19)	*P* Value
Age (year)	72.9 ± 7.5	68.6 ± 9.0	NS
Gender	11M13F	10M9F	NS
SBP (mmHg)	132.0 ± 17.9	136.7 ± 20.1	NS
DBP (mmHg)	79.0 ± 11.2	83.4 ± 12.0	NS
HR (bpm)	63.3 ± 12.3	63.0 ± 9.7	NS
MMSE	22.3 ± 4.2	29.5 ± 0.8	< 0.05
Duration (year)	3.9 ± 1.6		NS
Smoking	0	0	NS
Hypertension	14	11	NS
Dyslipidemia	11	9	NS
Diabetes	3	2	NS

Results are presented as the mean ± standard deviation. Abbreviations: *AD*=Alzheimer’s disease; *DBP*=diastolic blood pressure; *CN*=cognitively normal; *HR*=heart rate; *MMSE*=Mini-Mental State Examination; *NS*=not significant; *SBP*=systolic blood pressure; *Hypertension*=subjects with controlled hypertension; *Dyslipidemia*=subjects with controlled high blood cholesterol level on cholesterol medications; *Diabetes*=controlled type 2 diabetes without diabetic retinopathy

The patients and CN controls were advised to avoid physical exercise for 24 h before imaging and not to ingest alcohol or alcohol-containing products before the study visit. The neurologists or trained research associates administered the mini-mental state examination (MMSE). Each subject had an ophthalmic examination by the investigator, and these examinations included best corrected visual acuity, stereo vision, color vision, intraocular pressure (IOP) measurements as well as a slit-lamp examination of posterior and anterior segments.

The Retinal Function Imager (RFI, Optical Imaging Ltd., Rehovot, Israel) and its applications have been reviewed substantially [16]. Briefly, the system is a fundus camera-based imaging system that applies a stroboscopic light source and possesses high-speed video recording [17–20]. The system tracks the red blood cells as the intrinsic motion contrast agent using high-speed photography and measures retinal blood flow velocity in the branches of the central retinal arteries and veins. The procedure is non-invasive, and no external contrast is used. The image acquisition is synchronized with the cardiac cycle using a finger probe to reduce the influence of the cardiac cycle on the measurement. The reproducibility of the measurement of the blood flow was reported to be about 10% [21]. Using the information of vessel diameter and flow velocity, the macular blood flow supplying the macula can be obtained subsequently [7, 9].

The study protocol was the same as in our previous study [7]. The pupil was dilated with 1% tropicamide. Before imaging, the subject relaxed for 15 min in a room with dimmed light. The 20-degree field of view (FOV) was the first choice for the setting; because of its higher lateral resolution third, branches of the retinal vessel were more visible. Therefore, a FOV of 4.3 × 4.3 mm^2^ with a setting of 20 degrees centered on the fovea was used for imaging the macular region [7, 9]. As the 20-degree FOV has a relatively shallower focal depth and requires the subject to have steady fixation during fine adjustment of the focus, the 35-degrees FOV with a FOV of 7.3 × 7.3 mm^2^ was chosen as an alternative FOV. The larger FOV has a deeper focal depth, which makes it relatively easy to obtain a sharp focus on the retinal vessels. Both FOVs were applied in clinical research in previous studies [9, 16, 22]. In this study, the first choice was the FOV setting of 20-degrees, and the second choice was the FOV setting of 35-degrees. When the subject had difficulty on steadily fixating on the target, and sharp focusing was not achieved, the large FOV (35-degrees) was used [7]. A conversion factor as used in a previous study [7], was used to convert the blood flow measurements from the 35-degree FOV to the 20-degree FOV for analysis. The conversion factor was 0.95 for the BFV in the arterioles and 0.92 for the BFV in the venules, based on a previous comparison using both FOVs [23].

**Fig. 2:**
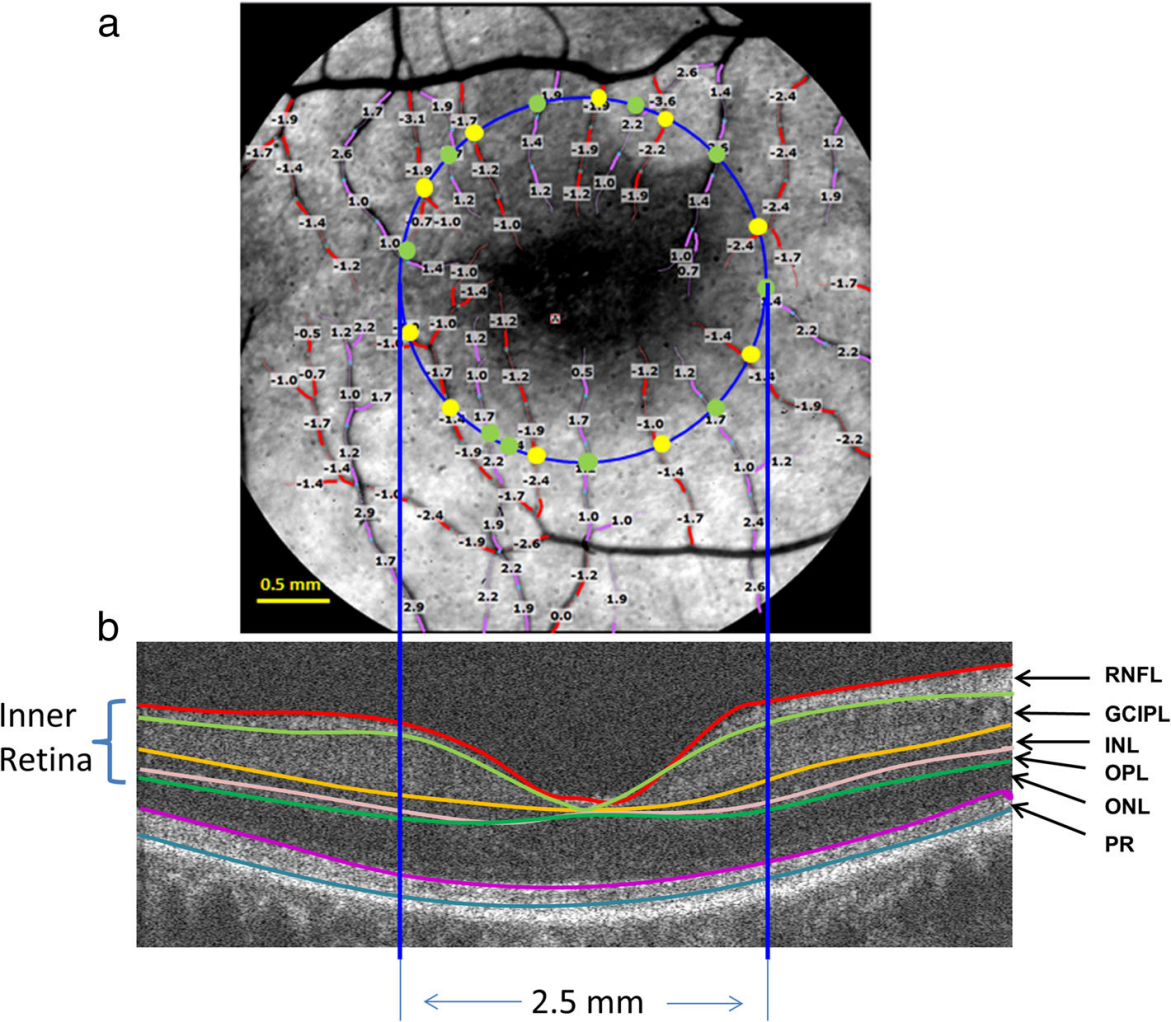
Retinal blood flow and tissue volume of the inner retina. **a**. A representative image of the retina from an AD patient was imaged using the RFI with a FOV of 4.3 × 4.3 mm^2^ (20-degrees setting) centered on the fovea, corresponding to the dark area in the center. The blood flow velocities (mm/s) were overlaid with the vessels. The arterioles marked in red have negative velocity values, indicating that blood is flowing away from the heart (flow moving towards the fovea). The venules marked in purple have positive velocity values, indicating that blood is flowing towards the heart. To analyze the blood flow in the macula, a 2.5 mm circle (blue) centered on the fovea was outlined. Vessel diameters of the vessels crossing the circle were measured at the locations marked as yellow and green dots. The arteriolar blood flow of these arterioles was calculated using the velocity and diameter (yellow dot). The venular blood flow of these venules was also calculated using velocity and diameter (green dot). All the flow measurements in the arterioles (all yellow dots) were summed to obtain the total arteriolar flow of the macula. Similarly, all the flow measurements in the venules (all green dots) were summed to obtain the total venular flow of the macula. **b**. To measure the tissue volume, the same eye was imaged using a custom UHR-OCT with a raster scan of 6 × 6 mm^2^. To calculate the tissue-dependent blood perfusion, volumetric tissue volume of the inner retina, including RFNL, GCIPL, INL and OPL was measured using segmentation software (Orion, Voxeleron) in the round area with a diameter of 2.5 mm centered on the fovea. RNFL: retinal nerve fiber layer; GCIPL: ganglion cell-inner plexiform layer; INL: inner nuclear layer; OPL: outer plexiform layer; ONL: outer nuclear layer; and PR: retinal photoreceptor

At least four well-focused images from each imaging session were obtained, and 3–5 sessions were acquired for the measurement of retinal blood flow. In one session, manual marking of the vessels was performed using the proprietary software, Browse (ver. 2.2.0.236). Thereafter, the software automatically registered all vessels in another selected session and obtained an average of vessel velocities from all sessions. BFVs of all the measurable vessels were calculated (Fig. 2). In tandem with previous studies [7, 9], the blood flow supplying the 2.5 mm diameter area centered on the fovea was quantified. Furthermore, the blood flows in the arterioles (supplying the flow into the area) and venules (moving the flow away from the area) are approximately equal [7, 9]. The diameter of the vessels that crossed the 2.5 mm circle was measured. In the intensity profile, perpendicular to the centerline of the vessel, the vessel diameter was obtained by counting the pixels of the full width and half of the maximum. The measurement was done using a custom software program developed in Matlab (Mathworks Inc., Natick, MA) [9]. The blood flow of each vessel segment crossing the circle was calculated [7, 9]. All flow rates in the arterioles or venules were summed to yield the total macular flow. Since arteriolar flow and venular flow are assumed to be approximately equal [7, 9], the macular blood flow was calculated as the average of arteriolar and venular flow volumes.

To measure retinal tissue volume, custom ultra-high resolution optical coherence tomography (UHR-OCT) was used. The system is a spectral domain OCT with a scan speed of 24,000 A-scans per second and an axial resolution of ~ 3 μm (in tissue) [24]. The volumetric data was acquired using a raster scan of 6 × 6 mm^2^ centered on the fovea. The dataset contained 128 consecutive B scans and 512 A-scans per B scan, and the scan depth was 2 mm. Image processing software (Orion, Voxeleron LLC, Pleasanton, CA, USA) was used to segment six intra-retinal layers and generate the volumetric measurement of each intra-retinal layer (Fig. 2). The segmented intra-retinal layers were RNFL, GCIPL, inner nuclear layer (INL), outer plexiform layer (OPL), outer nuclear layer (ONL), and retinal photoreceptor (PR). Retinal vasculature was found to span the inner retina from the RNFL to OPL [8, 25]. Therefore, to calculate retinal perfusion, the tissue volume including RNFL, GCIPL, INL, and OPL was considered as the tissue containing the retinal vasculature. The tissue in a diameter of 2.5 mm centered on the fovea was used to match the blood flow supply quantified in a 2.5 mm circle using the RFI. Tissue perfusion was calculated using the equation expressed as: tissue perfusion (nl/s/mm^3^) = blood flow (nl/s) / tissue volume (mm^3^).

A statistical software package (STATISTICA, StatSoft, Inc., Tulsa, OK) was used for descriptive statistics and data analysis. Student t-tests were used to analyze the differences in tissue perfusion between groups. To evaluate the relationship among the parameters that were not normally distributed, the Spearman rank-order correlation was used. A χ^2^ test was also used to analyze these categorical variables, which included confounding factors and gender. A result of *P <* 0.05 was considered statistically significant. Data are represented as mean ± standard deviation where appropriate.

## Results

**Fig. 3:**
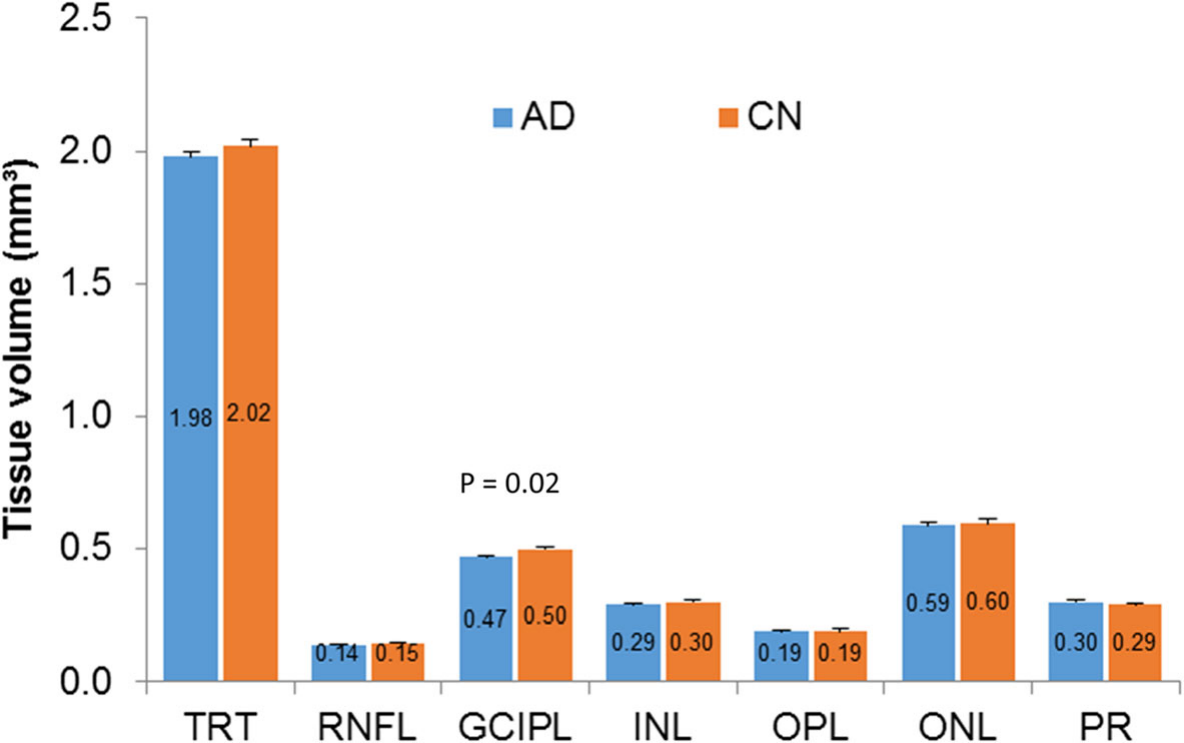
Tissue volumes of intra-retinal layers between the Alzheimer’s disease and cognitively normal groups. Among these intra-retinal layers and total retina, only GCIPL volume in the AD group was significantly lower (by 6%) than in the CN group (*P* < 0.05)

**Fig. 4:**
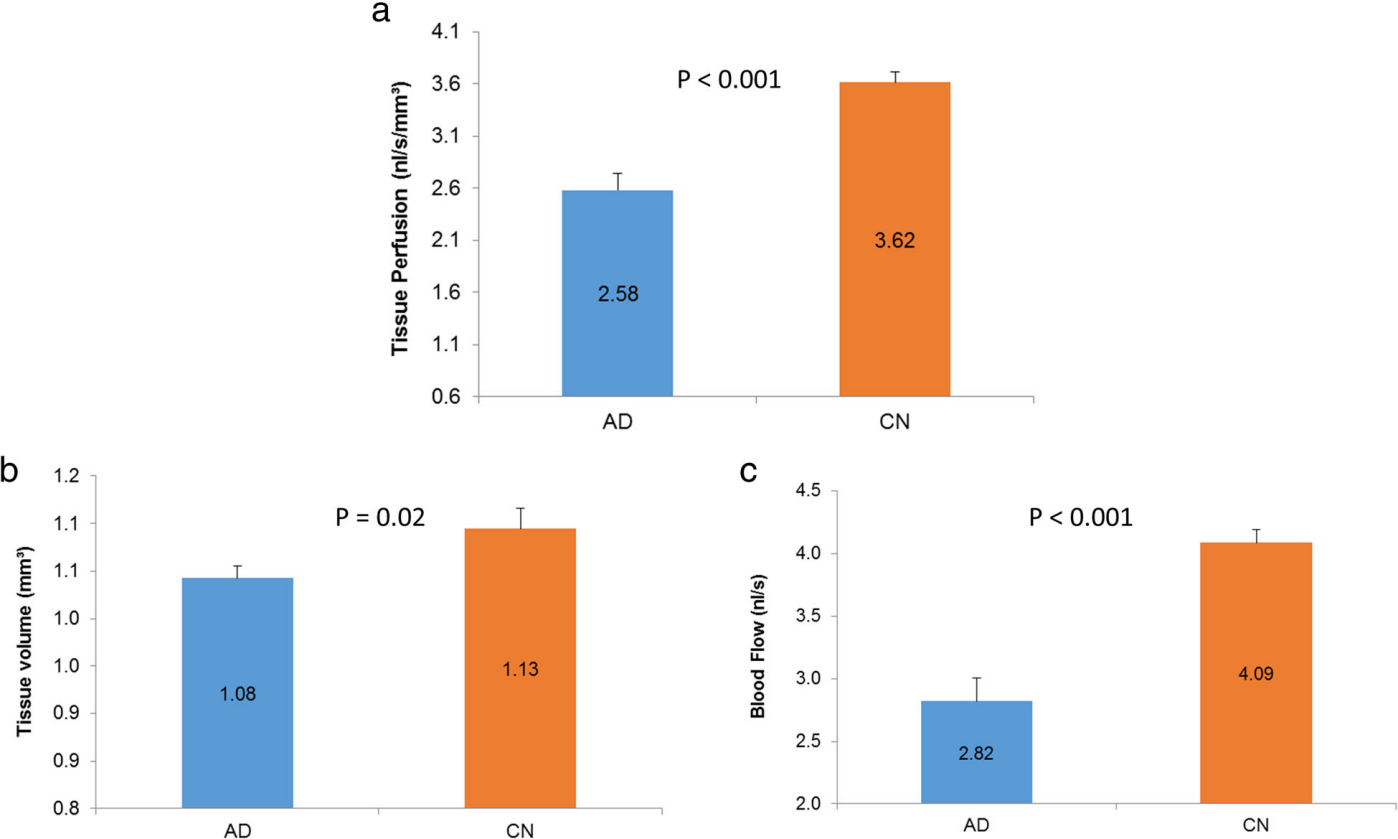
Tissue perfusion, tissue volume and blood flow in Alzheimer’s disease (AD) and cognitively normal (CN) groups. There were significant differences in tissue perfusion (**a**), tissue volume including RNFL, GCIPL, INL and OPL (**b**) and retinal blood flow (**c**) (*P* < 0.01)

The baseline characteristics of the participants are listed in Table 1. Among these intra-retinal layers and total retina, only GCIPL volume in the CAD group was significantly lower (by 6%) compared with the CN group (*P* = 0.02, Fig. 3). Tissue perfusion in the CAD group was 2.58 ± 0.79 nl/s/mm^3^, which was significantly lower than the CN group (3.62 ± 0.44 nl/s/mm^3^, *P* < 0.001, Fig. 4), reflecting a 29% decrease. Blood flow volume was 2.82 ± 0.92 nl/s in the CAD group, and was 31% lower when compared with the CN group (4.09 ± 0.46 nl/s, P < 0.001, Fig. 4). The tissue volume of the inner retina including RNFL, GCIPL, INL, and OPL was 5% lower in the CAD group compared with the CN group (*P* = 0.02).

**Fig. 5:**
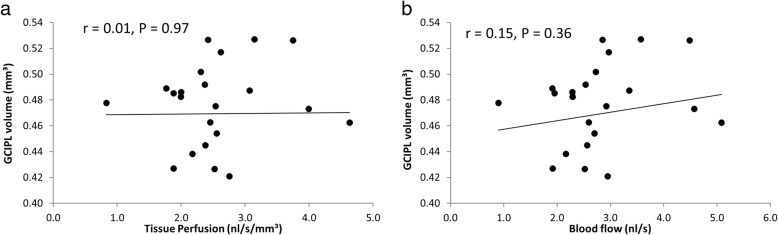
Correlation between GCIPL volume, tissue perfusion, and blood flow in CAD patients. GCIPL volume in CAD patients was neither related to tissue perfusion (*r* = 0.01, *P* > 0.05, **a**) nor blood flow (*r* = 0.15, P > 0.05, **b**)

MMSE and disease duration were not related to retinal tissue perfusion (*P* > 0.05) in AD. Tissue perfusion in the CAD group was not related to GCIPL (*r* = 0.01, *P* = 0.97, Fig. 5). Similarly, retinal blood flow was not related to GCIPL volume (*r* = 0.15, *P* = 0.36) in CAD patients.

## Discussion

To the best of our knowledge, this study is the first to quantify retinal tissue perfusion based on the direct measurements of the supplying blood flow and blood supplied tissue volume in the retina of CAD patients. The key finding is that CAD patients have macular tissue hypoperfusion that co-exists with GCIPL thinning. This finding agrees with the cerebral hypoperfusion previously measured in AD patients [26]. Cerebral perfusion is regarded as a key indicator of cerebral health [14]. The brain undergoes a wide array of anatomical and functional changes in normal aging [27], which is associated with an increased risk of age-related neurovascular and neurodegenerative diseases. These vascular changes affect cerebral tissue perfusion, compromise the functional integrity of the brain, and accelerate neurodegeneration in patients with AD.

As an age-related neurodegenerative disease, large-scale epidemiological studies have identified multiple vascular risk factors in AD patients, which contribute to the onset of the disease [28, 29]. Thus, controlling these vascular risk factors may prevent AD [30, 31]. Global and focal cerebral hypoperfusion are reported in AD, indicating the presence of brain tissue hypoperfusion [3–5]. Longitudinal studies have demonstrated that decreased cerebral perfusion in MCI could predict progression to AD [32]. In comparison to CN controls of similar ages, postmortem microscopic pathologic studies showed significant structural changes of the cerebral vasculature in AD, such as increased prevalence of microinfarcts, loose capillary density, capillary kinking, looping, and twisting [33]. These changes in the vessel systems may explain the impaired microcirculation, resulting in insufficient tissue perfusion. Image markers of large vessels measured with modalities such as MRI or TCD are helpful but mostly non-specific for assessing cerebral microvascular dysfunction. A non-invasive and inexpensive way to assess vascular alteration in the eye at a microvascular level, as performed in the present study, may also offer early diagnosis and subsequent monitoring of cerebral disease progression and efficacy of therapy.

Due to a shared anatomic origin and physiological similarities of vasculature between the brain and the retina, vascular changes in the retina may mimic changes in small cerebral vessels [34, 35]. In addition to previously documented impaired retinal blood flow velocity, flow rate and microvascular network [6, 7], this study provides the evidence of impaired retinal tissue perfusion in patients with AD, indicating diminished tissue metabolic activity in these patients. However, whether the impairment of tissue perfusion leads to or is a consequence of neural loss remains unresolved in this cross-sectional study. Further longitudinal studies are needed to address this question.

There are some limitations to the present study. First, this was a cross-sectional study that did not address whether tissue hypoperfusion leads to the loss of the tissue. Since retinal tissue perfusion was not related to GCIPL thinning, it could be speculated that tissue hypoperfusion may occur independent of neurodegeneration. Further longitudinal studies may validate this viewpoint of the time course of neurodegeneration and hypoperfusion. Second, the alterations of cerebral perfusion in patients with AD were not measured and therefore we could not compare the ocular and cerebral perfusion measurements. Third, two different FOVs were used in assessing blood flow velocity with the difference of approximately 8% between them in a previous study [23]. However, bear in mind that the decrease of tissue perfusion was ~ 30%, which was considerably larger than the measurement differences between the two FOVs.

## Conclusions

In conclusion, we report retinal tissue hypoperfusion and an association between decreased retinal blood flow and GCIPL thinning in patients with a clinical diagnosis of dementia of the Alzheimer type. Our findings may indicate diminished cerebral tissue metabolic activity together with microvascular changes in clinical Alzheimer’s disease.

## References

[1] World Health Organization (WHO) (2018). Dementia Fact Sheet.

[2] Corriveau RA, Bosetti F, Emr M, Gladman JT, Koenig JI, Moy CS (2016). The science of vascular contributions to cognitive impairment and dementia (VCID): a framework for advancing research priorities in the cerebrovascular biology of cognitive decline. Cell Mol Neurobiol.

[3] Hays CC, Zlatar ZZ, Wierenga CE (2016). The utility of cerebral blood flow as a biomarker of preclinical Alzheimer's disease. Cell Mol Neurobiol.

[4] Ruitenberg A, den Heijer T, Bakker SL, van Swieten JC, Koudstaal PJ, Hofman A, et al. Cerebral hypoperfusion and clinical onset of dementia: the Rotterdam Study. Ann Neurol. 2005;57(6):789–94. 10.1002/ana.20493 15929050

[5] Austin BP, Nair VA, Meier TB, Xu G, Rowley HA, Carlsson CM (2011). Effects of hypoperfusion in Alzheimer's disease. J Alzheimers Dis.

[6] Jiang H, Wei Y, Shi Y, Wright CH, Sun X, Gregori G, et al. Altered macular microvasculature in mild cognitive impairment and Alzheimer disease. J Neuroophthalmol. 2017; 10.1097/WNO.0000000000000580. 10.1097/WNO.0000000000000580 PMC5902666 29040211

[7] Jiang H, Liu Y, Wei Y, Shi Y, Wright CB, Sun X (2018). Impaired retinal microcirculation in patients with Alzheimer's disease. PLoS One.

[8] Campbell JP, Zhang M, Hwang TS, Bailey ST, Wilson DJ, Jia Y (2017). Detailed vascular anatomy of the human retina by projection-resolved optical coherence tomography angiography. Sci Rep.

[9] Jiang H, Delgado S, Tan J, Liu C, Rammohan KW, Debuc DC (2016). Impaired retinal microcirculation in multiple sclerosis. Mult Scler.

[10] Cheung CY, Ong YT, Hilal S, Ikram MK, Low S, Ong YL (2015). Retinal ganglion cell analysis using high-definition optical coherence tomography in patients with mild cognitive impairment and Alzheimer's disease. J Alzheimers Dis.

[11] Choi SH, Park SJ, Kim NR (2016). Macular ganglion cell -inner plexiform layer thickness is associated with clinical progression in mild cognitive impairment and Alzheimers disease. PLoS One.

[12] den Haan J, Janssen SF, van de Kreeke JA, Scheltens P, Verbraak FD, Bouwman FH (2018). Retinal thickness correlates with parietal cortical atrophy in early-onset Alzheimer's disease and controls. Alzheimers Dement (Amst).

[13] Bayhan HA, BayhanS A, Celikbilek A, Tanik N, Gürdal C (2015). Evaluation of the chorioretinal thickness changes in Alzheimer’s disease using spectral-domain optical coherence tomography. Clin Exp Ophthalmol.

[14] Chen JJ, Rosas HD, Salat DH (2011). Age-associated reductions in cerebral blood flow are independent from regional atrophy. Neuroimage.

[15] McKhann GM, Knopman DS, Chertkow H, Hyman BT, Jack CR, Kawas CH (2011). The diagnosis of dementia due to Alzheimer's disease: recommendations from the National Institute on Aging-Alzheimer's Association workgroups on diagnostic guidelines for Alzheimer's disease. Alzheimers Dement.

[16] Wang L, Jiang H, Grinvald A, Jayadev C, Wang J (2018). A mini review of clinical and research applications of the retinal function imager. Curr Eye Res.

[17] Landa G, Jangi AA, Garcia PM, Rosen RB (2012). Initial report of quantification of retinal blood flow velocity in normal human subjects using the retinal functional imager (RFI). Int Ophthalmol.

[18] Lopes de Faria JM, Andreazzi Duarte D, Larico Chavez RF, Arthur AM, Arthur R, Iano Y (2014). Reliability and validity of digital assessment of perifoveal capillary network measurement using high-resolution imaging. Br J Ophthalmol.

[19] Landa G, Rosen RB (2010). New patterns of retinal collateral circulation are exposed by a retinal functional imager (RFI). Br J Ophthalmol.

[20] Jiang H, Debuc DC, Rundek T, Lam BL, Wright CB, Shen M (2013). Automated segmentation and fractal analysis of high-resolution non-invasive capillary perfusion maps of the human retina. Microvasc Res.

[21] Burgansky-Eliash Z, Lowenstein A, Neuderfer M, Kesler A, Barash H, Nelson DA (2013). The correlation between retinal blood flow velocity measured by the retinal function imager and various physiological parameters. Ophthalmic Surg Lasers Imaging Retina.

[22] Landa G, Garcia PM, Rosen RB (2009). Correlation between retina blood flow velocity assessed by retinal function imager and retina thickness estimated by scanning laser ophthalmoscopy/optical coherence tomography. Ophthalmologica.

[23] Zhou J, Li M, Chen W, Yang Y, Hu L, Wang L (2017). Comparison of retinal microvessel blood flow velocities acquired with two different fields of view. J Ophthalmol.

[24] Tan J, Yang Y, Jiang H, Liu C, Deng Z, Lam BL (2016). The measurement repeatability using different partition methods of intra-retinal tomographic thickness maps in healthy human subjects. Clin Ophthalmol.

[25] Kur J, Newman EA, Chan-Ling T (2012). Cellular and physiological mechanisms underlying blood flow regulation in the retina and choroid in health and disease. Prog Retin Eye Res.

[26] Poels MM, Ikram MA, Vernooij MW, Krestin GP, Hofman A, Niessen WJ (2008). Total cerebral blood flow in relation to cognitive function: the Rotterdam Scan Study. J Cereb Blood Flow Metab.

[27] Morrison JH, Hof PR (1997). Life and death of neurons in the aging brain. Science.

[28] Ott A, Stolk RP, van Harskamp F, Pols HA, Hofman A, Breteler MM (1999). Diabetes mellitus and the risk of dementia: the Rotterdam Study. Neurology.

[29] Hofman A, Ott A, Breteler MM, Bots ML, Slooter AJ, van Harskamp F (1997). Atherosclerosis, apolipoprotein E, and prevalence of dementia and Alzheimer's disease in the Rotterdam Study. Lancet.

[30] de la Torre JC (2012). Cerebral hemodynamics and vascular risk factors: setting the stage for Alzheimer's disease. J Alzheimers Dis.

[31] de la Torre JC (2012). Cardiovascular risk factors promote brain hypoperfusion leading to cognitive decline and dementia. Cardiovasc Psychiatry Neurol.

[32] Park KW, Yoon HJ, Kang DY, Kim BC, Kim S, Kim JW (2012). Regional cerebral blood flow differences in patients with mild cognitive impairment between those who did and did not develop Alzheimer's disease. Psychiatry Res.

[33] Buée L, Hof PR, Delacourte A (1997). Brain microvascular changes in Alzheimer's disease and other dementias. Ann N Y Acad Sci.

[34] Patton N, Aslam T, MacGillivray T, Pattie A, Deary IJ, Dhillon B (2005). Retinal vascular image analysis as a potential screening tool for cerebrovascular disease: a rationale based on homology between cerebral and retinal microvasculatures. J Anat.

[35] London A, Benhar I, Schwartz M (2013). The retina as a window to the brain-from eye research to CNS disorders. Nat Rev Neurol.

